# Generalized Lichen Nitidus in Identical Twins

**DOI:** 10.1155/2012/982084

**Published:** 2012-11-22

**Authors:** Alexander K. C. Leung, Jeffrey Ng

**Affiliations:** ^1^Department of Pediatrics, The University of Calgary and The Alberta Children's Hospital, Calgary, AL, Canada T2M 0H5; ^2^Faculty of Medicine, The University of Calgary, Calgary, AL, Canada T2N 4N1

## Abstract

Lichen nitidus is an uncommon idiopathic chronic dermatosis characterized by minute, flesh-colored or hypopigmented, shiny papules commonly occurring on the abdomen, chest, genitalia, and extremities. The disorder is most often localized but, rarely, can become extensive or generalized. The occurrence of lichen nitidus during infancy is extremely rare. A perusal of the English literature revealed but two cases. We report two identical twins with lesions of generalized lichen nitidus noted at two months of age. The familial occurrence of lichen nitidus suggests that a genetic factor may be operative.

## 1. Introduction

 Lichen nitidus is an uncommon idiopathic chronic dermatosis characterized by minute, flesh-colored or hypopigmented, shiny papules commonly occurring on the abdomen, chest, genitalia, and extremities [1]. The disease was first described by Pinkus in 1907 [2]. In the majority of cases, the disease is localized. Rarely, cases of generalized lichen nitidus have been reported [1, 3]. Lichen nitidus most commonly affects children and young adults [1, 4]. The occurrence during infancy is very rare. We report two identical twins who had generalized lichen nitidus first noted at two months of age.

## 2. Case Reports

### 2.1. Case 1

KU, a Nigerian identical twin male infant, was born at 36 weeks' gestation, to a gravida 2, para 3, 31-year-old mother at term following a normal vaginal delivery. It was a twin pregnancy and this infant was the first born twin. The pregnancy was otherwise uncomplicated. The mother was not on any medication during the pregnancy. The parents were nonconsanguinous. Apgar scores were 9 and 9 at 1 and 5 minutes, respectively. The birth weight was 6 pounds and birth length was 19.5 inches. The neonatal course was uneventful and the past health was unremarkable. The infant presented with a generalized asymptomatic rash at two months of age. There was no history of any drug intake prior to the appearance of the rash. Except for the identical twin, there was no family history of a similar rash. 

**Figure 1:**
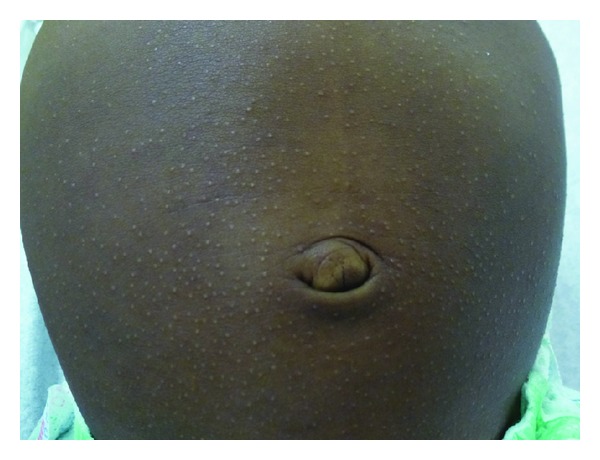
Numerous, discrete, flat-topped, hypopigmented, minute papules distributed on the abdomen of the first twin.

On examination, numerous, discrete, flat-topped, round, monomorphic, hypopigmented, shiny, pinhead-sized papules were noted on the abdomen (Figure 1), and to a lesser extent, chest, back, arms, legs, and buttocks. On the upper back, some of the lesions were grouped in linear arrays, possibly as a result of the koebnerization. The nails, palms, soles, and mucosal surfaces were normal. The remainder of the physical examination was normal. The clinical diagnosis of lichen nitidus was made. Parents were reassured of the benign nature of the condition and that treatment was not required.

### 2.2. Case 2

KQU, the second identical twin, was also delivered vaginally with no complication. His birth weight was 5 pounds 7 ounces and birth length was 19 inches. Agpar scores were 9 and 9 at 1 and 5 minutes, respectively. The neonatal course was uneventful. The infant was in good health and was not on any medication. The infant presented with a similar rash at 2 months. The rash was asymptomatic. 

**Figure 2:**
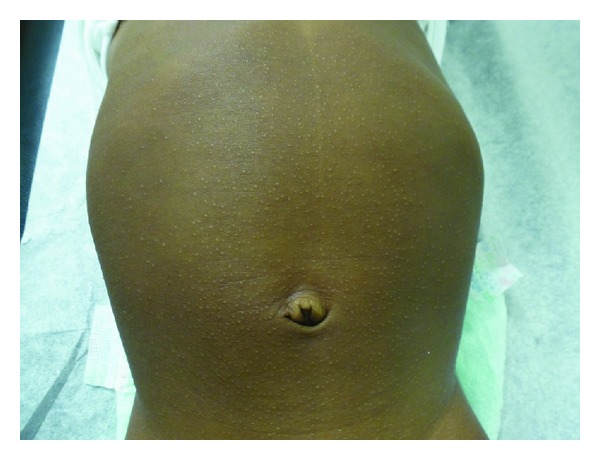
Numerous, discrete, flat-topped, hypopigmented, minute papules distributed on the abdomen of the second twin.

On examination, numerous, discrete, flat-topped, round, monomorphic, hypopigmented, shiny, papules measuring 1 to 2 mm were seen on the abdomen (Figure 2), chest, upper back, buttocks, arms, and thighs. These other affected areas were involved to a lesser extent than the abdomen. The nails, palms, soles, and mucosal surfaces were not involved. The remainder of the examination was unremarkable. Parents were reassured that the lesion of lichen nitidus would eventually resolve without sequelae and treatment was not required.

## 3. Discussion

 Clinically, lichen nitidus presents as minute, discrete, flat-topped, shiny papules, typically less than 3 mm in diameter [5]. Occasionally, the lesions can be dome-shaped [5]. Although the lesions are often flesh-colored, they may be hypopigmented in dark-skinned individuals, as is illustrated in the present cases [5]. The lesions are usually asymptomatic but may be pruritic [3, 5]. Sites of predilection include the chest, abdomen, genitalia, and extremities [3, 6]. Rarely, the palms, soles, nails, and mucous membrane may be involved [6]. The disorder is most often localized but, rarely, can become extensive or generalized as is illustrated in the present cases [1]. Evidence of koebnerization, with grouping of the papules in a linear array, may be seen [3, 5]. The diagnosis of lichen nitidus is mainly clinical, based on its distinctive features.

Lichen nitidus is an uncommon dermatosis. Data on the prevalence of this condition is very scarce. Hazen examined 11,729 Negro patients with skin diseases and found that 4 (0.034%) of them had lichen nitidus [7]. Lapins et al. reviewed the files of the Armed Forces Institute of Pathology in Washington, DC, USA and identified 43 cases of lichen nitidus [8]. Twenty one of these patients were Caucasian, 21 were Negro, and one was Spanish-American. Thirty four patients were male and nine patients were female. The patients ranged from 5 to 48 years of age. The 24 military male patients had a median age of 23 years, but only two of the 10 civilian patients were older than 10 years, their median age being seven years. The nine female patients had a median age of 13 years. Suffice to say, the majority of cases occur in children and young adults [4]. The occurrence of lichen nitidus during infancy is extremely rare. A perusal of the English literature revealed but two cases [9, 10]. Bercedo et al. reported a girl who had generalized lichen nitidus at the age of 11 months and who developed polyarticular juvenile chronic arthritis months later [9]. Rai and Singh described a one-month-old boy with lesions of lichen nitidus on his chin [10]. We report two identical twins with lesions of generalized lichen nitidus noted at two months of age. 

Familial lichen nitidus has rarely been reported [6, 11]. Marks and Jones reported two brothers, aged 10 and 12 years, with development of lichen nitidus within 2 to 3 days [11]. Kato described the occurrence of lichen nitidus in a 33-year-old father and his 3-year-old daughter [6]. We report the occurrence of lichen nitidus in two identical twins with onset at two months of age. Although the exact etiology is not known, the familial occurrence of lichen nitidus suggests that a genetic factor may be operative. It is possible that the genetic predisposition renders individuals susceptible to some environmental factors that induce lichen nitidus.

## References

[1] Al-Mutairi N, Hassanein A, Nour-Eldin O, Arun J (2005). Generalized lichen nitidus. *Pediatric Dermatology*.

[2] Pinkus F (1907). Uber eine neue knotchenformige hauteruption: lichen nitidus. *Archives of Dermatological Research*.

[3] Soroush V, Gurevitch AW, Peng SK (1999). Generalized lichen nitidus: case report and literature review. *Cutis*.

[4] Farshi S, Mansouri P (2011). Letter: generalized lichen nitidus successfully treated with pimecrolimus 1 percent cream. *Dermatology Online Journal*.

[5] Sanders S, De Collier AH, Scott R, Wu H, Scott McNutt N (2002). Periappendageal lichen nitidus: report of a case. *Journal of Cutaneous Pathology*.

[6] Kato N (1995). Familial lichen nitidus. *Clinical and Experimental Dermatology*.

[7] Hazen FH (1935). Syphilis and skin disease in the American Negro. *Archives of Dermatology and Syphilology*.

[8] Lapins NA, Willoughby C, Helwig EB (1978). Lichen nitidus. A study of forty-three cases. *Cutis*.

[9] Bercedo A, Cabero MJ, Garcia-Consuegra J, Hernado M, Yanez S, Fernandez-Llaca H (1999). Generalized lichen nitidus and juvenile chronic arthritis: an undescribed association. *Pediatric Dermatology*.

[10] Rai R, Singh DK (2008). Lichen nitidus. *Indian Pediatrics*.

[11] Marks R, Jones EW (1970). Familial lichen nitidus. The simultaneous occurrence of lichen nitidus in brothers. *Transactions of the St. Johns Hospital Dermatological Society*.

